# Bioinformatics approaches identified dasatinib and bortezomib inhibit the activity of MCM7 protein as a potential treatment against human cancer

**DOI:** 10.1038/s41598-022-05621-0

**Published:** 2022-01-27

**Authors:** Abdus Samad, Md. Amdadul Huq, Md. Shahedur Rahman

**Affiliations:** 1 Department of Genetic Engineering and Biotechnology, Jashore University of Science and Technology, Jashore, 7408 Bangladesh; 2 Bioinformatics and Microbial Biotechnology Laboratory, Department of Genetic Engineering and Biotechnology, Jashore University of Science and Technology, Jashore, 7408 Bangladesh; 3 Department of Food and Nutrition, College of Biotechnology and Natural Resource, Chung-Ang University, Anseong, Gyeonggi-do 17546 Republic of Korea

**Keywords:** Virtual drug screening, Cheminformatics, Pharmacodynamics, Pharmacokinetics

## Abstract

Minichromosome Maintenance Complex Component 7 (MCM7) is a key component of the DNA replication licensing factor and hexamer MCM (MCM2–7) complex that regulates the DNA replication process. The MCM7 protein is associated with tumor cell proliferation that plays an important role in different human cancer progression. As the protein is highly expressed during the cancer development process, therefore, inhibition of the protein can be utilized as a treatment option for different human cancer. However, the study aimed to identify potential small molecular drug candidates against the MCM7 protein that can utilize treatment options for human cancer. Initially, the compounds identified from protein-drugs network analysis have been retrieved from NetworkAnalyst v3.0 server and screened through molecular docking, MM-GBSA, DFT, pharmacokinetics, toxicity, and molecular dynamics (MD) simulation approach. Two compounds namely Dasatinib (CID_3062316) and Bortezomib (CID_387447) have been identified throughout the screening process, which have the highest negative binding affinity (Kcal/mol) and binding free energy (Kcal/mol). The pharmacokinetics and toxicity analysis identified drug-like properties and no toxicity properties of the compounds, where 500 ns MD simulation confirmed structural stability of the two compounds to the targeted proteins. Therefore, we can conclude that the compounds dasatinib and bortezomib can inhibit the activity of the MCM7 and can be developed as a treatment option against human cancer.

## Introduction

Nowadays cancer has been remarked as the most life-threatening disease in the world. Global Cancer Observatory (GLOBOCAN) a tool that predicts future cancer incidence, reported that 19.3 million new cancer cases in 2020 and almost 10.0 million cancer-related deaths worldwide^1^. Among them, the most deadly cancers were lung cancer that affects 1.8 million people around the world and causes deaths approximately 18%. Where colorectal, liver, stomach, and female breast cancer took around 9.4%, 8.3%, 7.7%, and 6.9% live worldwide^1^. The mortality rate is still increasing around the world due to aging and populations^2,3^. Despite extensive research and improvement of therapeutic approaches, the treatment of cancer is often diagnosed at a late stage. Early-stage detection of human cancer can improve the therapeutic option resulting decrease in the mortality rate^4,5^. Therefore, it is urgent to develop an effective therapy or drug candidates against different human cancer.

MCM7 is a part of the DNA replication licensing factor and hexamer MCM (MCM2–7) complex that regulates DNA replication^6–9^. The hexameric MCM protein forms a double trimeric complex along with the MCM4 and MCM6 that help to the unwinding of the DNA strand resulting initiation of DNA replication^8,10^. The frequency of chromosome breaks going to higher in cells under the replication stress due to the suppression of the MCM complex^8^. Downregulation of any one of its subunits destabilized the MCM complex and cells undergo limited replication. During the process, cells become hypersensitive to DNA replication stresses resulting in DNA damage and further inhibition of cell growth via activating the checkpoint signals^8,11^. Recently researcher has been found that MCM7 regulates the binding activity of MCM proteins that are highly associated with tumorigenesis and promotes cancer progression^12–15^. The MCM7 mRNA expression is a prognostic biomarker in ovarian, lung, and colorectal cancer and a tremendous biomarker in cervical cancer^12^. As the MCM7 overexpression plays a crucial role in cancer development, the study aimed to identify potential drug candidates against the protein to treat human cancer.

The conventional process of developing new drugs usually involves lengthy, expensive, and requires intense effort^16,17^. On the contrary, the computational design of a drug is relatively easier, requires low time and less effort^18^. The in silico virtual screening process help to generate lead compounds in the way of faster time and a lower cost^19^. Moreover, the computer-aided drug design (CADD) by using virtual screening process includes docking, absorption, distribution, metabolism, and excretion (ADME), toxicity, and molecular dynamics (MD) simulation has been applied to identify a diverse range of promising drug candidates^20^.

Recently researchers have been identified trichostatin A (TSA) as a promising drug candidate against human glioblastoma^21^, gastric, ovarian, breast, cervical, small-cell lung, and gastric cancer cell^22^. TSA is not just a possible inducer of apoptosis yet in addition engaged in the regenerated ability of stem cells^23^. Therefore, the study aimed to computationally discover potential drug candidates targeting the protein.

Initially, the study assess the protein-drugs interaction. Then targeting the protein potential drugs candidates has been identified by using the virtual screening, molecular docking, molecular mechanics-generalized born surface area (MM-GBSA), density functional theory (DFT), ADMET, and dynamic simulation approaches.

## Results

### MCM7 protein interaction with drugs or chemicals

**Figure 1:**
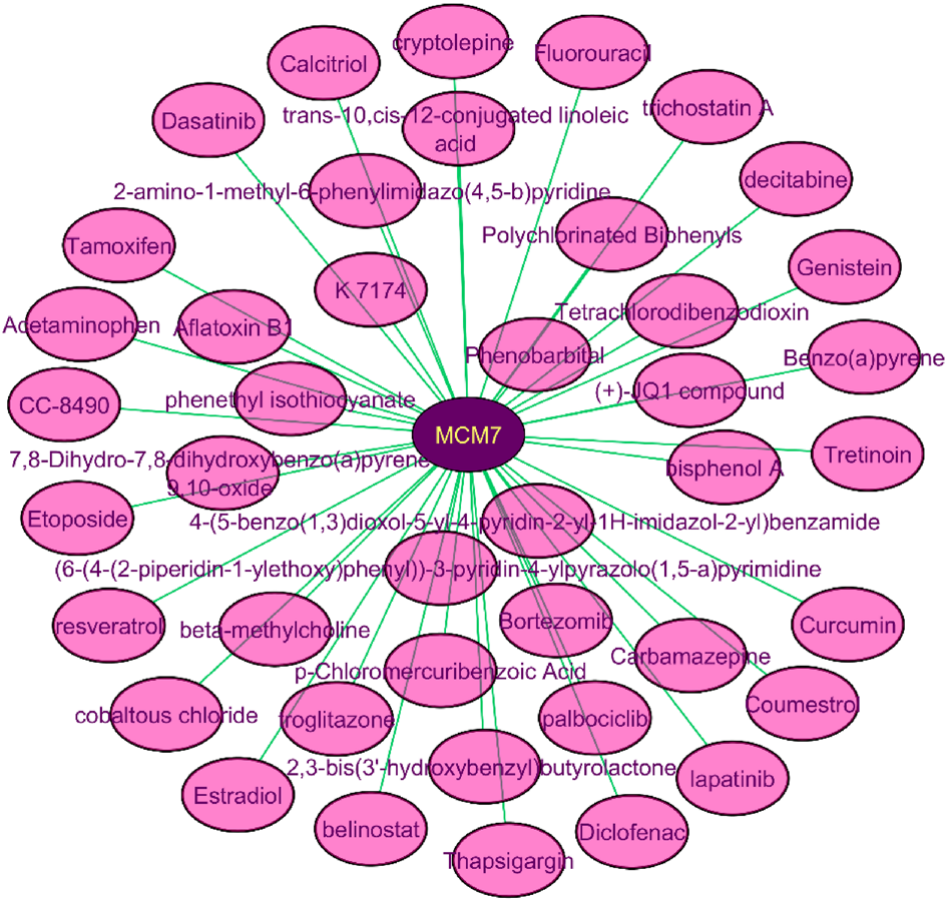
The protein–drugs interaction network of MCM7, where purple color represents the MCM7 protein and pink color represents the drugs.

The protein-drug interaction of all the MCM7 proteins was mapped out through the NetworkAnalyst website. A total of 41 compounds were found as a potential inhibitor against MCM7 in human cancer (Fig. 1) and their compound identification number (CID) has been retrieved from the PubChem website in SDF file format (SM 1).

### MCM7 protein-chemical compounds binding scores and MM-GBSA studies

**Figure 2:**
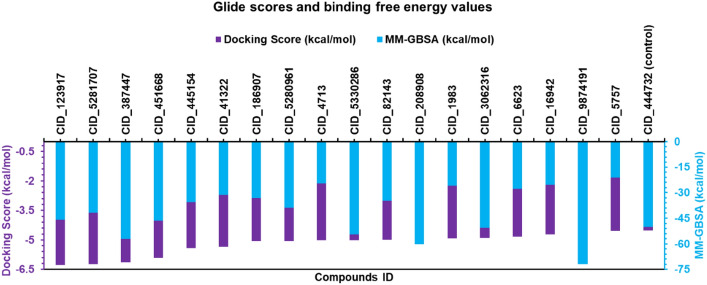
Bar graph representing the docking score and binding free energy values of the 18 hit compounds along with the control compound TSA.

Molecular docking between targeted protein and retrieved 41 ligand molecules has been performed by the glide v-8.8 tool. We find a total of 18 compounds having the highest binding affinity against MCM7 protein compared to the control ligand (TSA). Among 18 ligands CID_123917 shows high negative binding affinity and all docking scores range between − 6.265 and − 4.536 kcal/mol and the control (TSA) ligand binding score was − 4.529 kcal/mol (Fig. 2 and SM 2). High 18 binding scores containing ligands were selected for MM-GBSA analysis compared to control ligand (TSA). In the MM-GBSA calculation, CID_9874191 and CID_5757 produced the negative highest and lowest MMGBSA ΔG Binding (NS) score of − 71.85 kcal/mol and − 21.25 kcal/mol. Among 5 ligands was showed a good binding free energy score (− 71.85 to − 50.65 kcal/mol) compared to control (− 50.11 kcal/mol) (Fig. 2 and SM 2). Top 5 and control compounds based on their docking and MM-GBSA score were chosen for further evaluation.

### MM-GBSA studies of selected compounds

**Figure 3:**
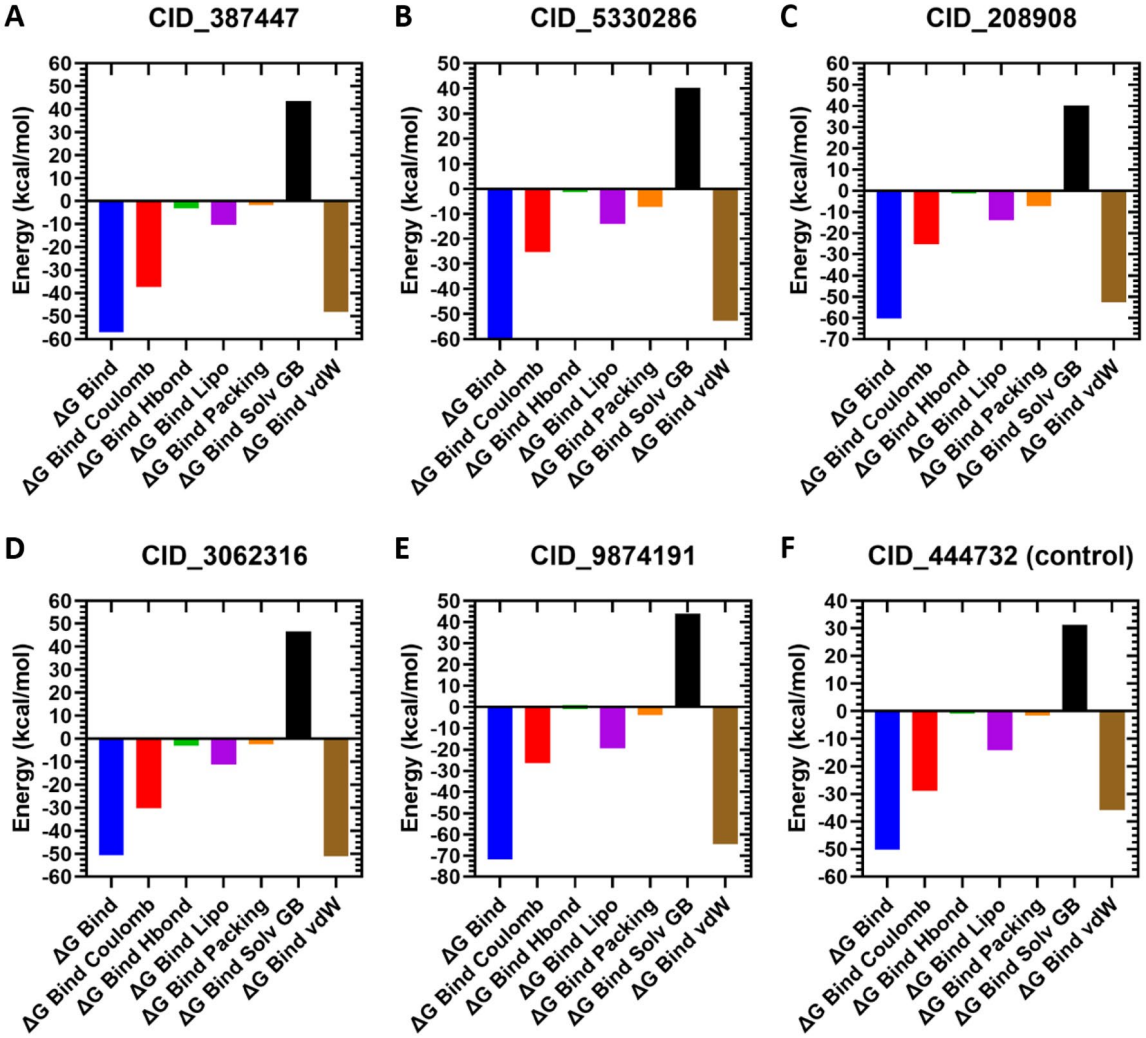
MM-GBSA binding free energy values of the selected docked compounds were comparison with TSA.

MM-GBSA calculation of the selected compounds CID_9874191, CID_208908, CID_387447, CID_5330286, CID_3062316, and CID_444732 (control) produced the negative MMGBSA ΔG Bind(NS) score of − 71.85, − 60.32, − 56.97, − 54.41 to 71.85, − 50.65, and − 50.11 kcal/mol, respectively. Furthermore, examination of binding free energy values for each MCM7-ligands docked complexes significantly exposed the performance of ΔGBind Coulomb (Coulomb energy), ΔGBind H-bond (Hydrogen bond energy), ΔGBind Lipo (Lipophilicity energy), and ΔGBind vdW (Van der Waals interaction energy) in the respective complex stability. These outcomes measured the strong binding affinity of target compounds such as CID_9874191, CID_208908, CID_387447, CID_5330286, CID_3062316, by comparison to CID_444732 (control) of human MCM7 protein (Fig. 3). The top five compounds include control compounds have been chosen based on their docking and MM-GBSA score and rederived for further analysis.

### Ligand optimization by QM (quantum mechanical) calculation

**Figure 4:**
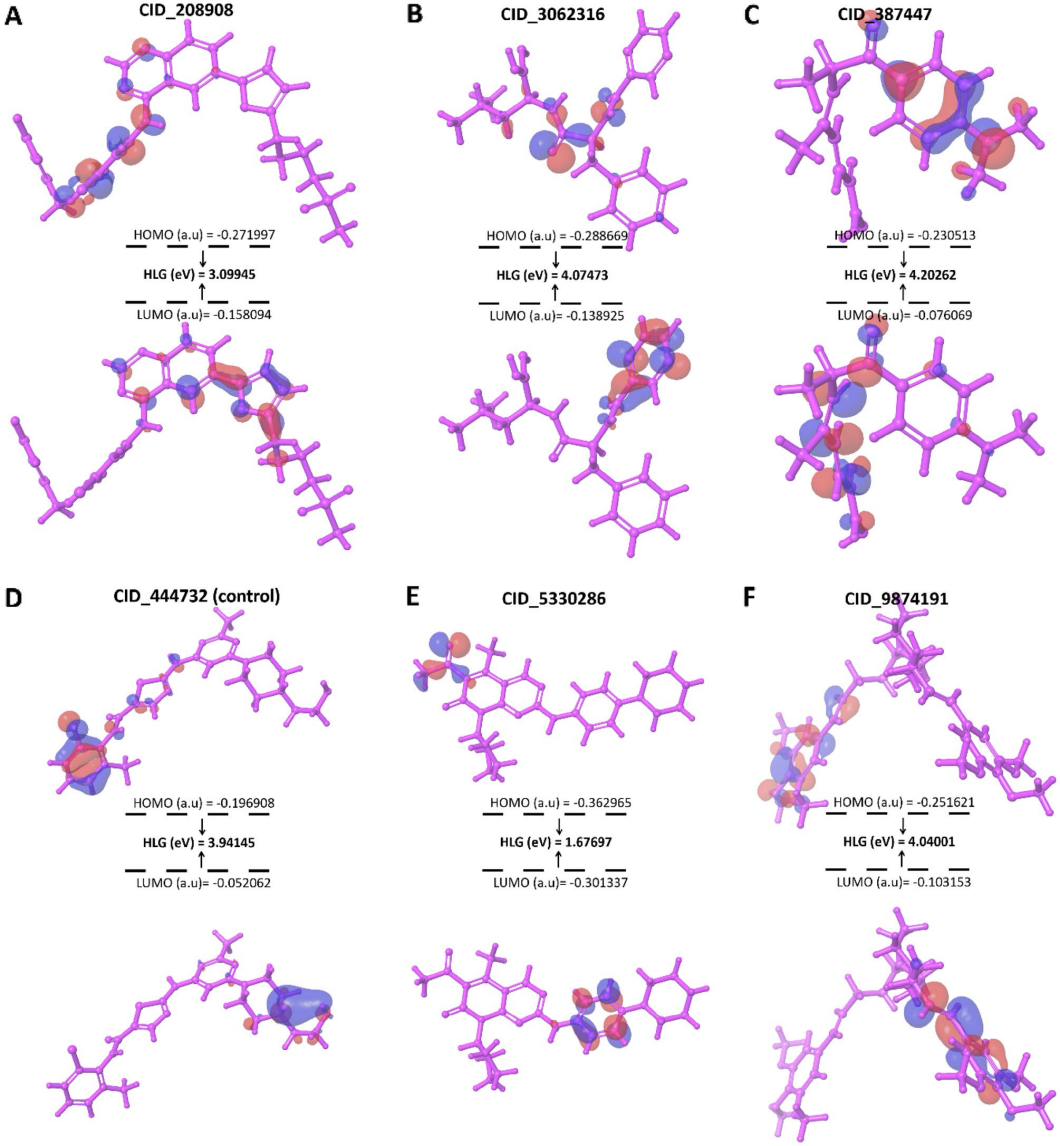
The HOMO and LUMO energy score and structure of the 6 docked compounds were compared with TSA.

In the DFT calculations, CID_9874191, CID_208908, CID_387447, CID_5330286, CID_3062316, and CID_444732 (control) generated a HOMO and LUMO energy score of − 0.26642 and − 0.1060, − 0.19453 and − 0.06287, − 0.23891 and − 0.07207, − 0.28103 and − 0.14699, − 0.21012 and − 0.05364, and − 0.19369 and − 0.04438 a.u (Fig. 4 and Table 1).

**Table 1:** DFT calculation of the selected compounds after molecular and MM-GBSA analysis.

Pubchem ID	HOMO (a.u)	LUMO (a.u)	HLG (eV)	Hardness (eV)	softness (eV)
CID_387447	− 0.23891	− 0.07207	4.20262	2.10131	0.4758
CID_5330286	− 0.28103	− 0.14699	1.67697	0.83847	1.1926
CID_208908	− 0.19453	− 0.06287	3.09945	1.549725	0.6452
CID_3062316	− 0.21012	− 0.05364	4.07473	2.037365	0.4908
CID_9874191	− 0.26642	− 0.10601	4.04001	2.020005	0.4950
CID_444732 (control)	− 0.19369	− 0.04438	3.94145	1.970725	0.5074

CID_387447 and CID_5330286 generated HLG, hardness, and softness energy of 4.20262 and 1.67697, 2.10131 and 0.83847, and 0.4758 and 1.1926 eV, respectively (Table 1). Also, CID_208908 and CID_3062316 produced the HLG, hardness, and softness energy of 3.09945 and 4.07473, 1.549725, and 2.037365, and 0.6452 and 0.4908 eV, respectively (Table 1). On the other hand, CID_9874191 and CID_444732 (control) gave HLG, hardness, and softness energy of 4.04001 and 3.94145, 2.020005 and 1.970725, and 0.4950 and 0.5074 eV, respectively (Table 1). CID_387447, CID_3062316, and CID_9874191 compounds were selected compared with CID_444732 (control) compound for further analysis.

### Molecular features of the selected chemical compounds

ADMET properties are one of the valuable parameters for designing effective drug candidates against a specific target. Drug likeness properties of selected compounds have been accessed according to the Lipinski rule of five (RO5). The selected five compounds have the highest negative binding affinities and obtained the RO5. ADMET properties of selected compounds have been presented in Table 2. According to the SwissADME and PKCSM server, all the selected ligands have low toxicity, no Ames toxicity, good GI absorption, good distribution rate, good water solubility (logS), and logP value expect for the compounds CID_9874191 which has a high molecular weight (Table 2).

**Table 2:** Molecular features of the selected chemical compounds.

Drug properties	CID_387447	CID_3062316	CID_9874191	CID_444732 (control)
**Physicochemical properties**				
MW (g/mol)	384.24	488.01	568.74	302.37
Heavy atoms	28	33	41	22
Arom. heavy atoms	12	17	12	6
Rotatable bonds	11	8	16	7
H-bond acceptors	6	6	8	3
H-bond donors	4	3	0	2
**Lipophilicity**				
Log Po/w	0.22	2.8	5.15	2.37
**Water solubility**				
Log S (ESOL)	− 2.71	− 4.98	− 6.41	− 3.19
**Pharmacokinetics**				
GI absorption	High	High	High	High
BBB permeant	No	No	No	No
**Drug likeness**				
Lipinski, Violation	Yes,0	Yes,0	Yes,1	Yes,0
**Toxicity**				
AMES toxicity	No	No	No	No
Oral Rat Acute Toxicity (LD50) (mol/kg)	1.868	2.676	2.986	1.897
Skin Sensitization	No	No	No	No

### Visualization of selected chemical compounds-MCM7 protein

**Figure 5:**
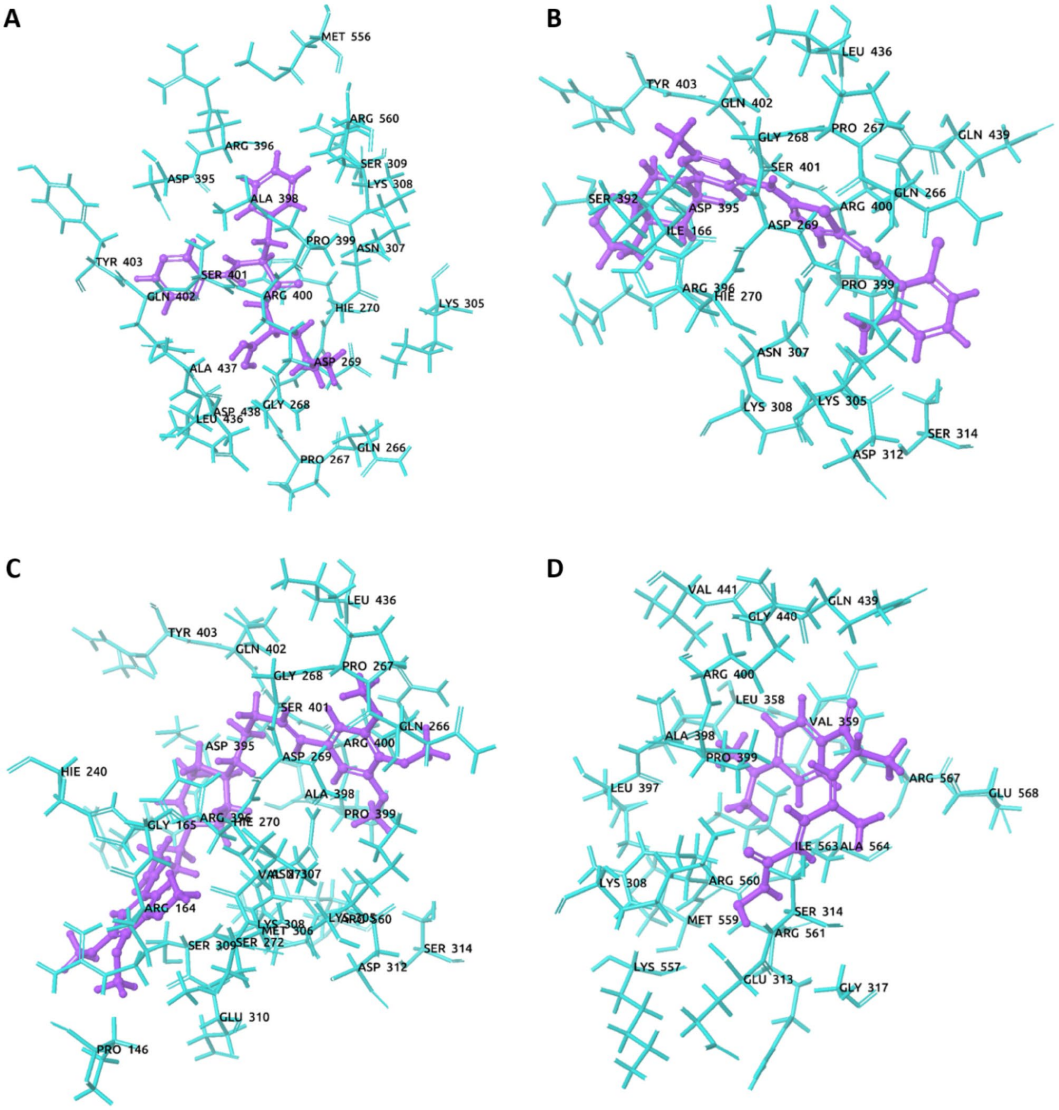
The 3D interaction between MCM7 protein and selected compounds complex structure. (A) CID_387447, (B) CID_3062316, (C) CID_9874191, and (D) CID_444732 (control).

The best docking, MM-GBSA, DFT, and ADMET score containing selected five compounds (CID_387447, CID_3062316, CID_9874191, and CID_444732 (control)) has been retrieved for further analyzed and molecular interactions have been visualized through Maestro v12.5 (Fig. 5, Table 3, and SM3). Different types of non-bonded interactions between receptors and ligands like hydrogen bonds, electrostatic bonds, and hydrophobic bonds have been identified. Among the selected drugs, CID_387447 showed the − 6.137 kcal/mol binding affinities with MCM7 and interacted H-bond binding residues at Gly268, Leu436, Ser401, and Asn307 residue position (Fig. 5 and Table 3). The molecule of the binding affinities (− 4.905 kcal/mol) has CID_3062316 formed three hydrogen bonds with the receptor and interacted binding residues was Asp395, Gly268, and Lys305 (Fig. 5 and Table 3). The CID_9874191-receptor complex (binding affinity: − 4.578 kcal/mol) was stabilized with two hydrogen bonds (Lys305 and Ser401). CID_444732 (control)-receptor complex having a binding energy of − 4.529 kcal/mol was stabilized with one hydrogen bond (Lys308). The binding interactions of top molecules have presented in Table 3 and illustrated in Fig. 5.

**Table 3:** A list of various interactions and interacting residues of MCM7 with the compounds were logged from respective docked complexes.

Compounds	H-Bond	Other interaction
CID_387447 (Bortezomib)	Gly268, Leu436, Ser401, and Asn307	Tyr403, Gln402, Arg400, Pro399, Ala398, Arg396, Asp395, Lys308, Asn307, Lys305, Gln266, Asp269, Hie270, Ala437, and Arg560
CID_3062316 (Dasatinib)	Asp395, Gly268, and Lys305	Ile166, Hie270, Asp269, Gln266, Asn307, Lys308, Ser314, Asp312, Tyr403, Gln402, Ser401, Arg400, Pro399, and Arg396
CID_9874191 (K-7174)	Lys305 and Ser401	Met306, Asn307, Lys308, Ser309, Arg560, Arg164, Gly165, Hie240, Asp395, Arg396, Ala398, Pro399, Arg400, Gln402, Ser272, Val271, Hie270, Asp269, Gly268, Pro267, Gln266, and Leu436
CID_444732 (control)	Lys308	Gly440, Gln439, Ser314, Glu313, Leu358, Leu397, Ala398, Pro399, Glu368, Arg567, Ala564, Ile563, Arg561, Arg560, Ser314, and Glu313

### Stability of MCM7 protein-chemical compounds

**Figure 6:**
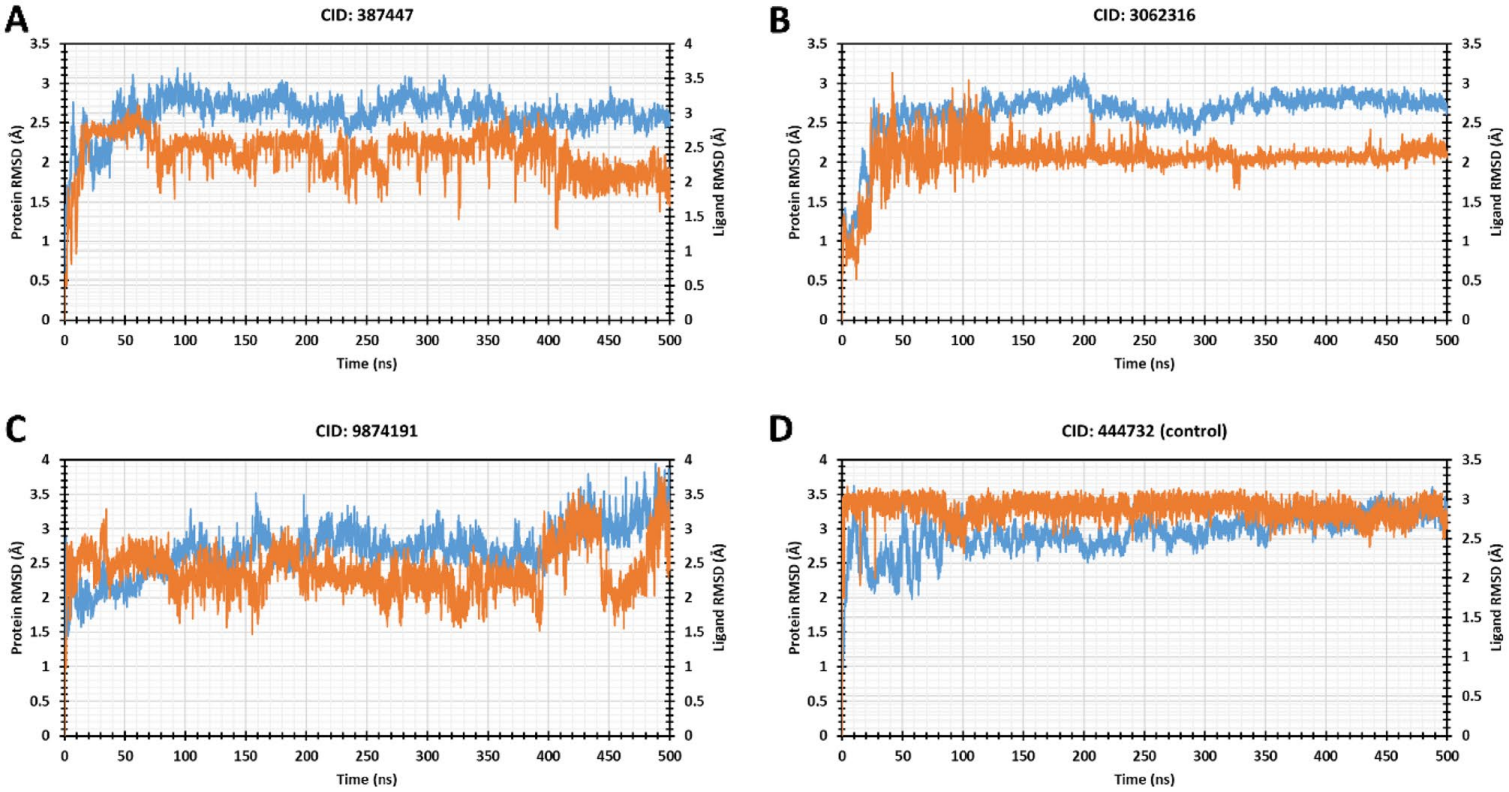
Graphs represent the MD simulation for the selected protein-ligands complex during 500 ns simulation time. Herein, representing the compounds (**A**) CID_387447, (**B**) CID_3062316, (**C**) CID_9874191, and (**D**) CID_444372 RMSD values of MCM7 protein (blue curves) and ligands (red curves).

The root means square deviations (RMSDs) of Cα atoms have been computed for the compounds CID_387447, CID_3062316, CID_9874191, and CID_444732 (control) protein–ligand complexes to measure the protein structure stability throughout the 500 ns simulation time. The compound having a CID_387447 complex becomes stable after 370 ns then it shows very low fluctuation 0.365 Å was found during 307–500 ns simulation time. The average, highest, and lowest RMSD value was found with a value of 2.641 Å, 3.196 Å, and 0.685 Å during 500 ns simulation time. Where the ligand average RMSD value was 2.404 Å during the 500 ns simulation time, which is the 2nd best compound compared to the rest of the three compounds (Fig. 6A). However, the lowest RMSD was 1.2 Å during the 150 ns simulation time. The compounds CID_3062316 complex show stability after 307 ns simulation time, where its average fluctuation of RMSD 0.331 Å was found during 307–500 ns simulation time and the average protein and ligand RMSD was 2.638 Å and 2.049 Å. Herein, the compound with CID_3062316 also shows a good RMSD and the best lowest difference RMSD between protein and ligand complex system (Fig. 6B). During the whole simulation time, the CID_9874191 shows the highest and average RMSD (3.950 Å and 2.699 Å) and it is quite unstable and the average fluctuation was 0.833 Å during the 410 ns to 500 ns. The compound CID_9874191 also shows an unacceptable RMSD value and very high fluctuation (Fig. 6C). However, 444,372 (Control) complexes become stable at 100–132 ns but it fluctuates (0.492 Å) found higher after 110 ns time. At the end of the simulation, it shows 2.941 Å average RMSD which value is high compared to CID_3062316 and CID_387447 (Fig. 6D). However, the compounds CID_3062316 and CID_387447 show less fluctuation among all the four selected compounds shown in Fig. 6.

**Figure 7:**
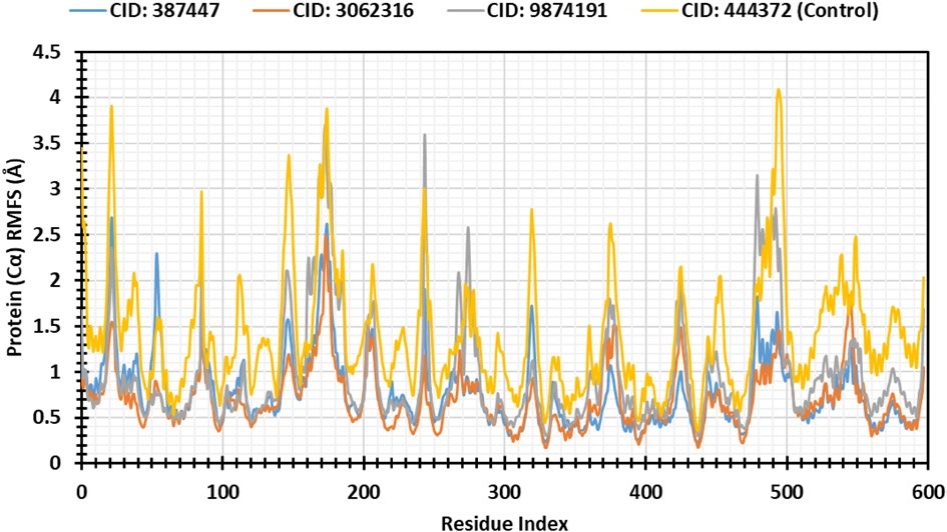
Graphs exhibiting the information about the RMSF values of MCM7 protein by 500 ns time of MD simulation.

Root mean square fluctuation (RMSF) indicate the fluctuation of amino acid (AA) residue in protein structure. The high RMSF value of the amino acid residues determines the fluctuation and stability level of AA residues in a complex system. Initially, the compound CID_9874191 complex shows the highest amino acid fluctuation 3.703 Å, where all the complexes are bound to < 3 Å RMSF value. Secondly, the compound CID_444372 (Control) shows that the highest RMSF value of 4.088 Å. CID_387447 and CID_3062316 complex was showing the highest amino acid fluctuation of 2.682 Å and 2.489 Å. During the RMSF analysis, the compounds CID_3062316 and CID_387447 show the highest stability compared to control compounds depicted in Fig. 7.

**Figure 8:**
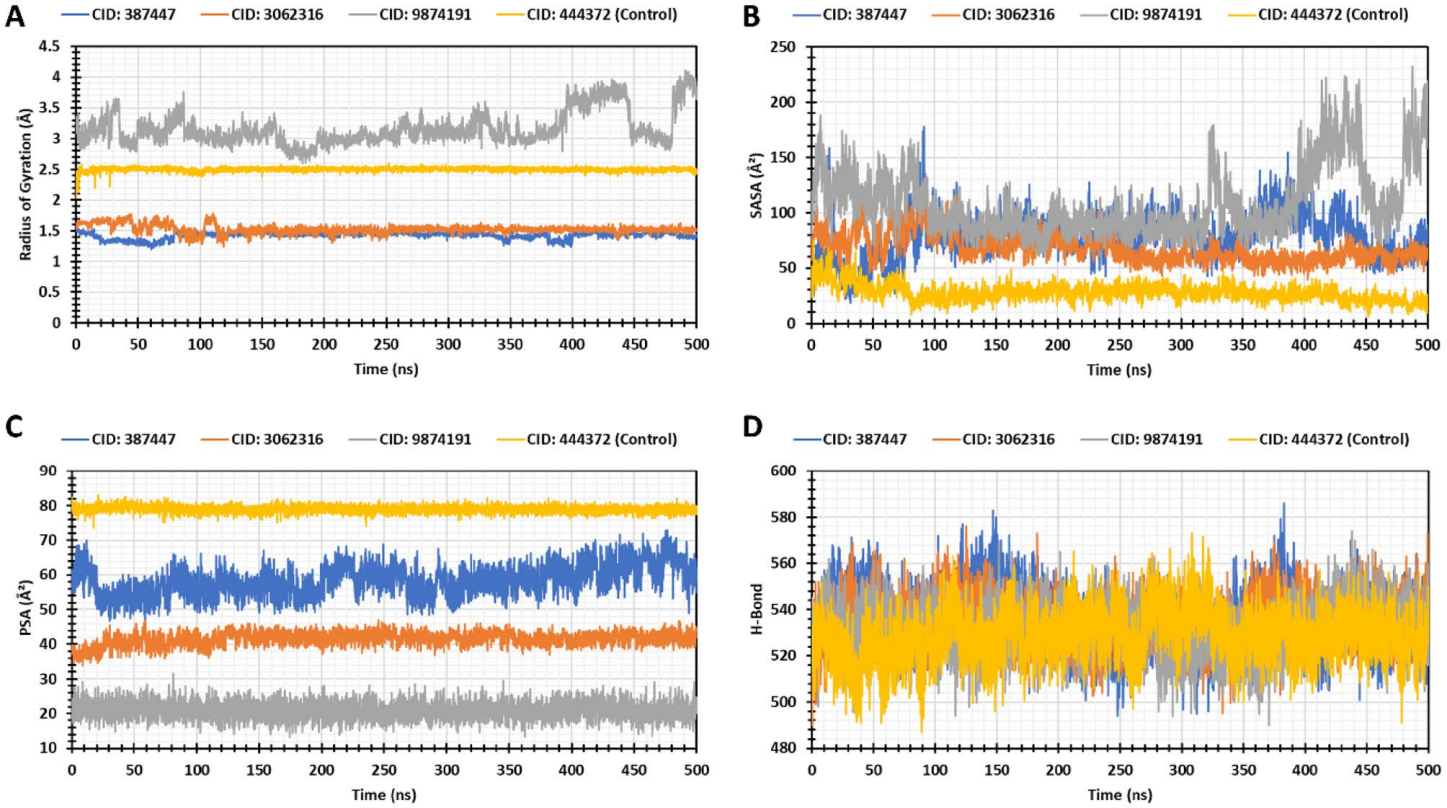
Graphs represent the MD simulation result by 500 ns time. Here, (**A**) exhibited the result of the radius of gyration (Rg) values of MCM7 protein, (**B**) denote the values of SASA, (**C**) PSA, and (**D**) Hydrogen bonds.

To determine the protein mobility and rigidity, the radius of gyration (Rg) of the protein–ligand complex has been analyzed. The compounds CID_9874191 and CID_444372 (control) exhibited the maximum and minimum trend of fluctuations range 9.128 Å to 5.776 Å (difference 3.352 Å) and 5.199 Å to 4.078 Å (difference 1.121 Å). The CID_3062316 and CID_387447 showed the minimum and maximum fluctuations rate of 4.459 Å to 6.372 Å (difference 1.913 Å) and 3.372 Å to 4.521 Å (difference 1.149 Å), and both compounds were more stable compared control ligand (Fig. 8A).

Additionally, the Solvent Accessible Surface Area (SASA), Polar Surface Area (PSA), Hydrogen Bond (H-Bond) of the compounds have been analyzed for all the proteins and protein-drug complexes. SASA analysis is useful in understanding the solvent-like behavior (hydrophilic or hydrophobic) of a protein molecule as well as protein–ligand complexes. The compounds CID_387447, CID_3062316, CID_9874191, and CID_444372 exhibited the minimum and maximum trend of fluctuations range 507.866–53.001 Å^2^, 140.411–459.061 Å^2^, 135.384–560.323 Å^2^, and 11.671–150.234 Å^2^ (Fig. 8B). The PSA and hydrogen bond analysis of CID_3062316 and CID_387447 found the compounds have good stability (Fig. 8C,D). The results suggested that both CID_3062316 and CID_387447 protein-drug complexes were impressively stable after the binding of drug molecules.

### Protein–ligand contact analysis

**Figure 9:**
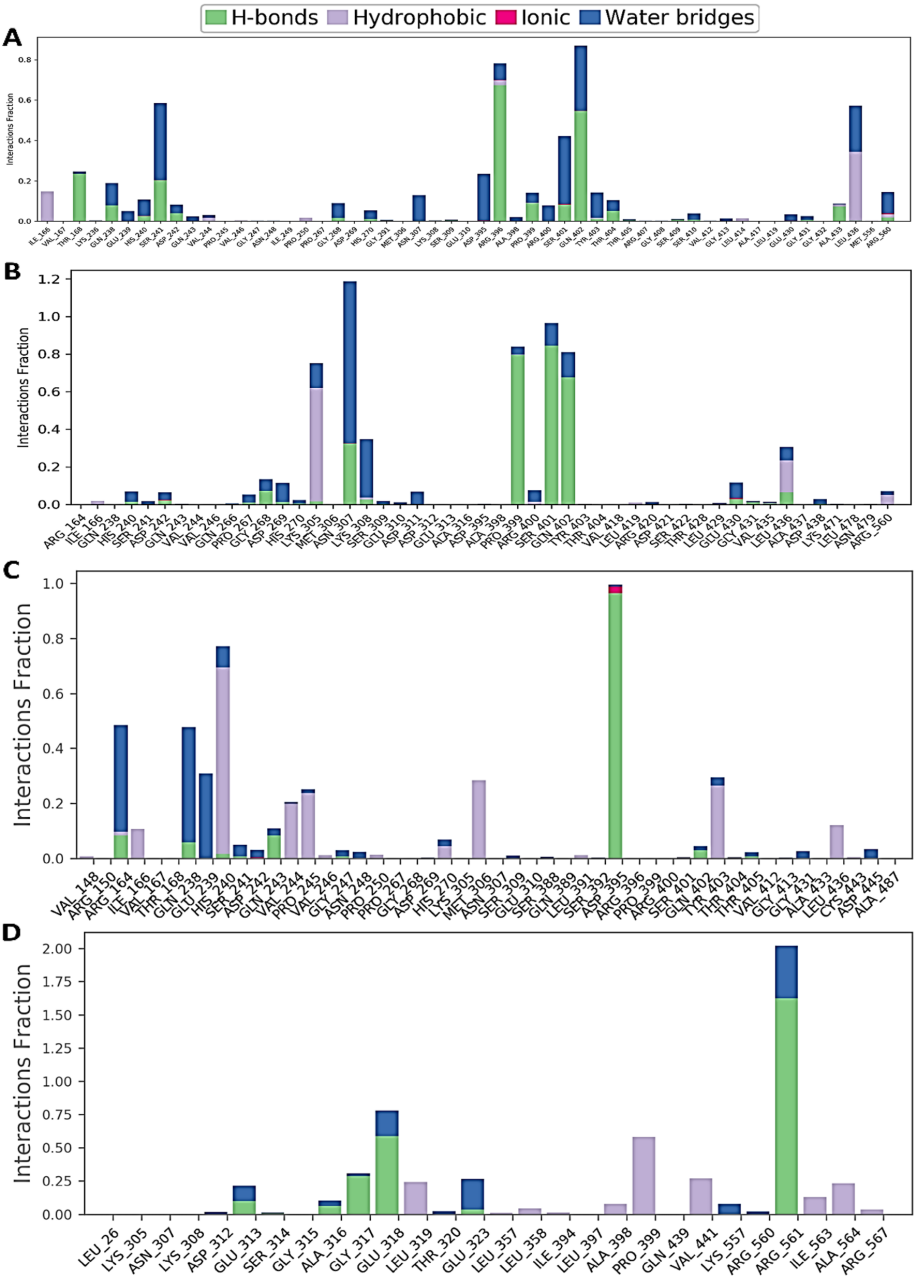
Graphs exhibiting the information about the protein–ligand interaction by 500 ns time of MD simulation. Herein, representing the compounds (**A**) CID_387447, (**B**) CID_3062316, (**C**) CID_9874191, and (**D**) CID_444372.

The complex structure of the protein with the selected ligands and, their intermolecular interactions has been evaluated at 500 ns simulation time via the 'simulation interactions diagram (SID). Depending on some parameters including hydrogen bond, non-covalent bond (hydrophobic bond), ionic bond, and water bridges bond the contact between protein and ligands complex structure includes CID_387447, CID_3062316, and CID_9874191 have been analyzed and represented in Fig. 9. The compound CID_387447 generated multiple (more than two) interactions at THR168, SER241, ARG396, PRO399, SER401, GLN402, and LEU436 residues with an interaction fraction (IF) value 0.3, 0.6, 0.8, 0.18, 0.5, 0.9, and 0.6 simulation time the specific interaction is maintained by the multiple contacts of the same subtype with the ligand accordingly (Fig. 9A). The compound CID_3062316 formed multiple interaction at LYS305 (0.8), ASN307 (1.2), PRO399 (0.9), SER401 (1.0), GLN402 (0.8), and LEU436 (0.4) residues maintained by simulation time accordingly (Fig. 9B). CID_9874191 compounds formed multiple interaction at ARG150 (0.5), THR168 (0.5), HIS240 (0.8), ASP395 (1.0), SER401 (0.1), and GLN402 (0.3), residues maintained by simulation time accordingly (Fig. 9C). In the case of the compound CID_444372, it has found to form multiple interactions at the position of GLU318 (0.8), PRO399 (0.6), and ARG561 (2.0) suggests that of the simulation time the specific interaction is maintained and helped to make a stable binding with the desired protein (Fig. 9D). However, the compounds CID_3062316 and CID_387447 show good hydrogen and other bond interaction with all the four selected compounds shown in Fig. 9.

### Ligand–protein contact analysis

**Figure 10:**
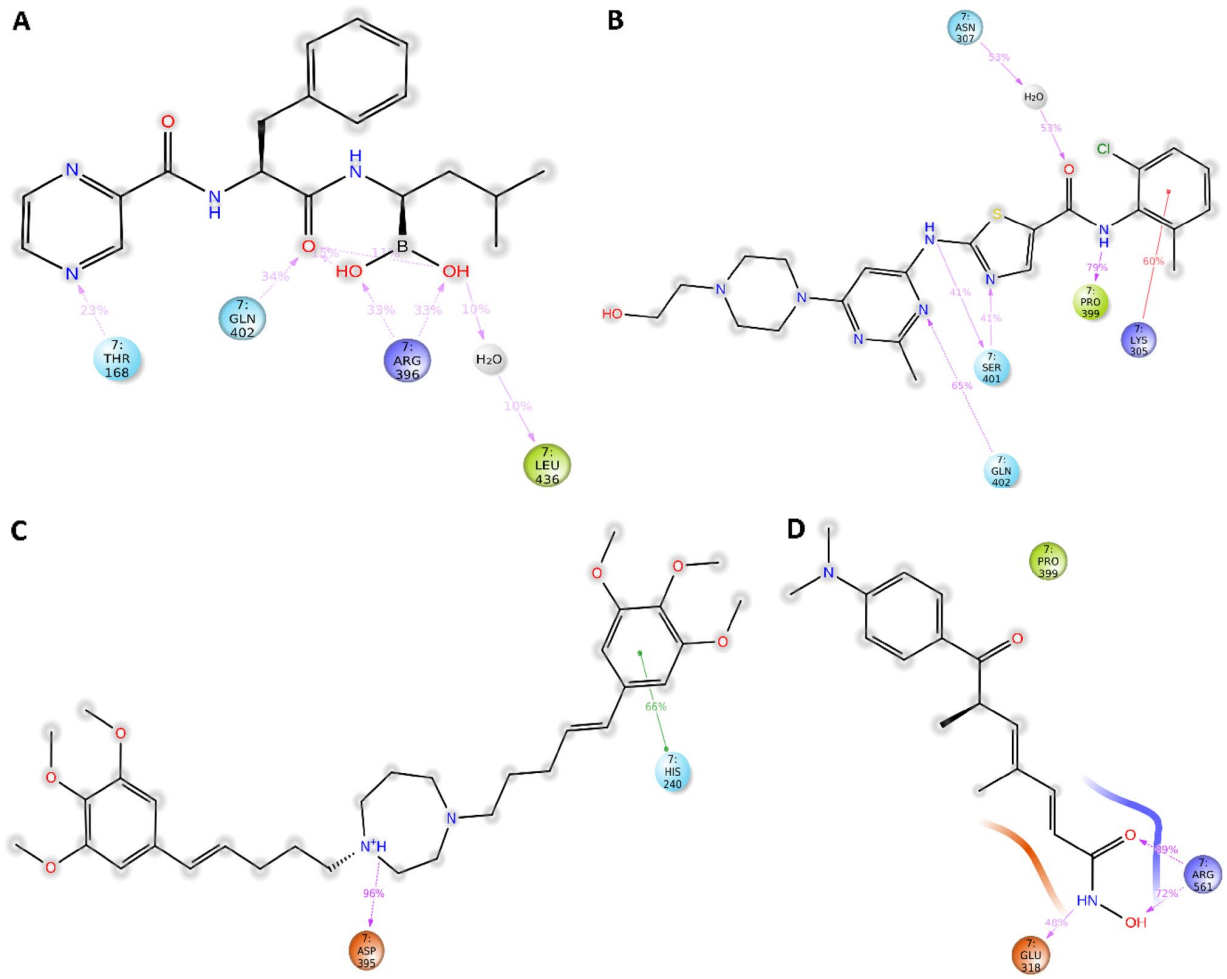
Graph exhibiting the information about the ligand–protein contacts, after 500 ns simulation. In this place, (**A**) CID_387447, (**B**) CID_3062316, (**C**) CID_9874191, and (**D**) CID_444732 (control).

The selected three ligands CID_387447, CID_3062316, CID_9874191, and CID_444372 (control) with protein interactions have been monitored throughout the SID. The compound CID_387447, CID_3062316, CID_9874191, and CID_444372 (control) generated multiple (more than two) interactions residues of the simulation time the specific interaction is maintained by the multiple contacts of the same subtype with the ligand accordingly (Fig. 10). In ligand–protein interaction analysis, CID_3062316 and CID_387447 were both compounds that are more stable compared to control compounds (Fig. 10).

### MM-GBSA analysis from post molecular dynamic simulation trajectory

**Figure 11:**
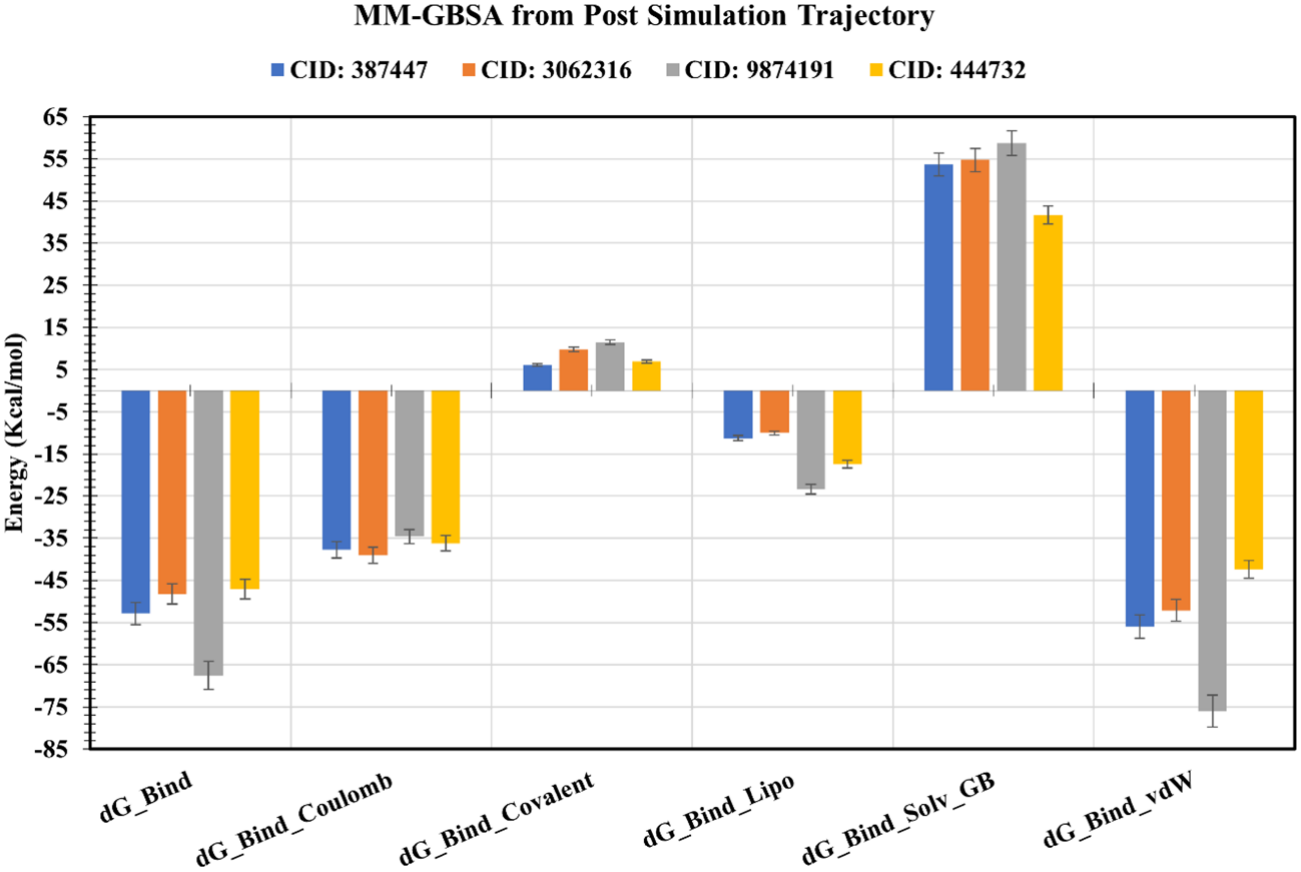
Representing different energy components and net MM/GBSA binding free energy (kcal/mol) and standard deviation values generated from extracted snapshots of MCM7 protein in complex with selected compounds.

MM/GBSA methods have been used in this study to estimate the ligand-binding free energy to the desired protein. The MM/GBSA of the protein–ligand complex structure has been calculated from the few snapshots (∼ 500 ns) of the dynamic simulation trajectory. The analysis of the complex structure found higher net negative binding free energy values − 52.851 kcal/mol, − 48.241 kcal/mol, − 67.541 kcal/mol, and − 47.107 kcal/mol for the selected four compounds CID: 387,447, CID: 3,062,316, CID: 9,874,191, and CID: 444,732, respectively with the targeted protein (Fig. 11). Therefore, it can be considered that the selected compounds will be able to maintain a long-term interaction with the desired MCM7 protein.

## Discussion

Cancer is the subsequent driving reason for the highest mortality around the world. Generally, the pervasiveness of disease has expanded compared to the previous era^24,25^. In this manner, malignant growth is a major issue influencing the wellbeing of humans. Shockingly, it is an assortment infection at the tissue level and this assortment is a significant test for its particular treatment, trailed by the ability of therapy^26,27^. The MCM7 association in human cancer significantly immunostaining for human cancers: colorectal adenocarcinoma, endometrial carcinoma, esophageal adenocarcinoma, melanoma cancer, oral squamous cell carcinoma, thyroid cancer, and glioblastoma^28^. In this study, we demonstrated that MCM7 mRNA expression was markedly down or up-regulated in human cancer. To date our knowledge, this is the comprehensive computational analysis conducted for the identification of potential drugs like candidates against human cancer by targeting the MCM7 protein.

Computer-Aided Drug Design (CADD) is one of the most promising tools for the selection of novel compounds against a specific protein as it includes different advanced features and techniques^29^. The CADD approach has minimized the required time and costs involved in the entire drug discovery process that makes the virtual screening process includes molecular docking, MM-GBSA, DFT, MD simulation, and ADMET, etc. as integral parts of drug designing^30^.

In this study, we identified MCM7 and their potential drug candidates by molecular docking and other processes. Initially, the molecular docking process has used to screen the compounds, where the top 18 compounds have been selected with the highest negative binding affinities compared to the control ligand (TSA). All the selected ligands have higher negative binding affinities than previously reported TSA compounds related to human cancer^21,22^. In the MM-GBSA study, the most negative ΔGBind score (the lowest score) is considered as the best ΔGBind score^31^. Analysis of MM/GBSA found higher net negative binding free energy values for the five selected and control compounds in complex with MCM7 protein. Thus, the remaining compound has skipped and the five selected compounds CID_387447, CID_5330286, CID_208908, CID_3062316, and CID_987419 along with TSA (CID_444732) has been taken for further evaluation through the DFT based calculation.

Frontier orbitals study or DFT calculation is one of the essential methods determining the pharmacological properties of various small molecules^31,32^. HOMO and LUMO help to determine and understand the chemical reactivity and kinetic stability of small molecules. The difference between HOMO and LUMO energy is known as HOMO–LUMO gap energy corresponds to the electronic excitation energy. The compound that has the greater orbital gap energy, tends to be energetically more unfavorable to undergo a chemical reaction and can be called bioactive^33,34^. Moreover, gap energy also correlates with the hardness and softness properties of a molecule^35^. The DFT calculations were carried out for all the three best ligand molecules compared to the TSA control ligand. Thus, two molecules have been eliminated during the different stages of screening and the remaining three compounds CID_387447, CID_3062316, and CID_9874191 with TSA (CID_444732) were selected for ADMET analysis.

The RO5 demonstrated the drug-like properties of the selected compounds^36,37^. All the three compounds CID_387447, CID_3062316, and CID_9874191 with control TSA ligand (CID_444732) were found to follow the five Lipinski’s rules of drug-likeness properties. The compound with good ADME properties has been further evaluated through the toxicity properties to measure the harmful effect on humans or animals^38^. Analysis of toxicity found no or less toxicity of the selected three compounds CID_387447, CID_3062316, and CID_9874191) along with TSA.

Molecular dynamics simulation is used to confirm the stability of a protein in a complex with ligands^30,38^. Also, it can determine the stability and rigidity of protein–ligand complexes in a specific artificial environment like the body^30^. The RMSD values of the complex systems indicate the best stability of the compounds and RMSF values measure mean fluctuation that determines the compactness of the protein–ligand complex^39^**.** The compounds CID_387447 and CID_3062316 showed lowest RMSD and RMSF values expect CID_9874191 compared to CID_444732 control compound. The center of mass from the protein C and N terminals tests the stability of the protein structure and gives a broader understanding of protein folding characteristics for Rg calculated^40^. The lower Rg value means that high compactness and the larger value displayed the disassociation of the compounds from the protein and all the compounds except CID_9874191 showed better Rg value. The larger SASA value indicates the less stable structure whereas the lower value means the tightly contracted complex of water molecules and amino acid residues^41^**.** Further, the evaluation of SASA values, hydrogen bond interaction, protein–ligand contact, ligand–protein contact found diverse results, therefore the compound CID_9874191 has been eliminated.

According to the researchers, Dasatinib (CID_3062316) inhibitors could be used to treat lung cancer^42^, gastrointestinal stromal tumors, prostate cancer, multiple myeloma, and sarcomas^43^. Therefore, Bortezomib (CID_387447) is a reversible proteasome inhibitor that impacts the ubiquitin–proteasome pathway to kill cancer cells, and proteasome inhibition modifies the transcriptional expression of many target genes^44,45^. The K-7174 (CID_9874191) could be a promising treatment option for chronic disease anemia^46^. The Dasatinib and Bortezomib drugs would be theoretically stable and capable of generating an effective inhibition response to MCM7 protein. Further assessment through various lab-based trial methods can assist with deciding the action of the compound that will give options in contrast to human cancer immunotherapy.

## Conclusion

An integrative protein-drug interaction, molecular docking, ADMET, QM calculation, MD simulation, and MM-GBSA approaches revealed CID_387447 and CID_3062316, as potential drug candidates that will help to inhibit the activity of the MCM7 protein against human cancer. Further assessment through various lab-based trial methods can assist with deciding the action of the compound that will give options in contrast to human cancer immunotherapy.

## Methods

In this study, we used the Linux (Ubuntu-20.04.1 LTS) operating system and Intel Core i7-10700 K processor CPU, 3200 MHz DDR4 RAM and RTX 3080 DDR6 8704 CUDA core GPU. Computational molecular docking, MM-GBSA, and DFT calculation were generated by Glide, Prime, Jaguar, and Maestro. The molecular dynamic simulation was performed by using the Desmond module of Schrödinger Suite 2020-3.

### Protein structure retrieval and preparation

The crystal structure of human MCM7 protein (PDB code: 6XTY) was downloaded from RCSB Protein Data Bank (PDB)^47^. MCM7 protein was co-crystalized with the MCM protein family, therefore the target MCM7 protein was separated and removed the water, metal ions, cofactors, other molecules, and other proteins by the Maestro v-12.5 of Schrödinger Suite 2020-3^48^. The MCM7 protein was initially processed and prepared by the protein preparation wizard (Prep Wizard) of Schrödinger Suite 2020-3^49^. The prepared protein has been further utilized for molecular docking and other experiments.

### Compounds identification and preparation

The MCM7 protein and its correspondence compounds interaction were performed by the NetworkAnalyst v3.0 server^50^ and the protein-drugs interaction network was redesigned using the Cytoscape v3.7^51^. Therefore, this interacting chemical compound’s structure has been searched on the PubChem website^52^ and retrieved in SDF file format. Finally, chemical compounds were processed, refined, and prepared by the LigPrep v-55139^53^ for molecular docking, MM-GBSA, and DFT calculation.

### Binding affinity calculation and MM-GBSA analysis

The desired protein-compounds binding scores have been evaluated by the molecular docking approach^54^. The best binding score of compounds and protein interaction has been analyzed and visualized by using the Glide v-8.8 and Maestro v-12.5.139 respectively^55,56^ (Schrödinger packaged 2020–3) tool^57^. The binding site position of the protein has been determined by reference ligand active site and a grid box corresponding to the binding site position has been generated. A grid box with a box shape X = 25.086 Å, Y = 25.086 Å, Z = 25.086 Å has been set for molecular docking simulation. Nowadays most of the lead compounds identified in the CADD process are based on the docking score (protein-chemical compounds binding scores), which does not always provide an accurate and constant score. Therefore, validation of the docking process through different energy calculation methods can provide the reliability of the methods^58^. MM-GBSA (molecular mechanics-generalized born surface area) was calculated to calculate the bind-free energy of ligands and validated the docking process. The MM-GBSA score was predicted by Prime MMGBSA v-3.0^59^. We selected the OPLS_2005 force field^60^ and other parameters were defaults. From this, the docking score and MM-GBSA score were obtained as a control to compare the value with newly screened drugs. Binding interactions, residues, and binding free energy involved in the interacting plane were analyzed with Maestro v-12.5.139^48^.

### QM (quantum mechanical) calculation

Conformation analysis of a ligand to the binding site of a protein is an essential part to identify potential active conformation, binding affinity, and strain discipline associated with the binding mechanism. This type of binding possess can be achieved through the calculation of minimum energy conformation and structural optimization, which is dependent on the solution phase and associated gas-phase energy. The classical molecular mechanics (MM) process is unable to describe the process properly due to the presentation of metal ions in a ligand–protein complex system^61^. The ligand was minimization by QM (quantum mechanical) calculations using the density functional theory (DFT), these performed by the Jaguar v-10.9^62^. The DFT was treated by B3LYP (Becke exchange functional^63^, which combined Lee, Yang, and Parrs (LYP)^64^ correlation functional) in conjunction with 6-31G(d,p) basis sets. For this reason, after the highest docking score and MM-GBSA calculated ligand was selected for DFT or QM calculation. In DFT calculation, we analyzed frontier molecular orbitals namely highest occupied molecular orbitals (HOMOs), lowest unoccupied molecular orbitals (LUMOs), and their energy gap difference. When HOMOs energy values show the ability of a ligand molecule to donate electrons, LUMOs energies propose the capability of a ligand molecule to accept electrons from the protein. The frontier energies (ε) of HOMOs and LUMOs were used to measure the hardness and softness of selected compounds. The hardness (η) and softness (s) of the drugs were measured performed by the Parr and Pearson interpretation equation ^65^ and Koopmans theorem equation^66^. The hardness value determines how the atom resists the charge transfer to another atom or metal surface. The ability of an atom to receive electrons is measured by the softness value. The following Equations can be used to measure the chemical hardness (1) and softness (2).

1 $${\text{Hardness}}\;\left( \eta \right) = \left( {{\text{I}} - {\text{A}}} \right)/{2}$$

2 $${\text{Softness}}\;\left( {\text{S}} \right) = {1}/\eta$$

In Equation, I refer to the ionization potential (− E_HOMO_). Also, A denotes the electron affinity (− E_LUMO_). According to the above-mentioned Equation, the smaller value of hardness means more reactivity and vice versa. Whereas S is the ability of an atom to receive electrons and η is the hardness.

### Visualization of selected chemical compounds-MCM7 protein

The constructed protein-compounds interaction network was redesigned using the Cytoscape v3.7^51^. The docking binding interactions and ligand DFT calculation figure were generated and visualized with Maestro v-12.5.139^48^. The docking score, MM-GBSA, and MD simulation graphs have been designed in GraphPad Prism 8 and Microsoft Excell365.

### Molecular features of the selected chemical compounds

The drug design and development process involves the assessment of ADME/T (absorption, distribution, metabolism, excretion, and toxicity) to identify molecules with the highest pharmacokinetics properties and can be an effective drug^16^. The integrity and efficiency of compounds should be described through pharmacokinetic and toxicity properties in the early stages of drug design. To assess the early-stage pharmacokinetic properties of our chosen compounds, the SwissADME (http://www.swissadme.ch/) server was used in study^67^. The SwissADME server, a free web-based tool that can analyze small molecule pharmacokinetics and drug-likeness properties. Toxicity evaluation is the crucial stage in the development and design of a drug. Therefore, the toxicity of the selected compounds has been evaluated through the pkCSM (http://biosig.unimelb.edu.au/pkcsm/) server^68^.

### Molecular dynamics simulation

The complex structure of the selected candidate compounds was evaluated using 500 ns MD simulation to evaluate their binding stability to the desired protein to the active site cavity of the protein^60^. The MD simulation of the receptor-ligand complex wereas performed using the ‘Desmond v6.3 Program' in Schrödinger 2020-3 under Linux framework to evaluate the thermodynamic stability of the receptor-ligand complexes^30^. To solve the system, a predetermined TIP3P water model was used, with an orthorhombic periodic boundary box form with a box distance of 10 Å assigned to both sides to retain a specific volume. Boundary conditions box volume was initially calculated as 910,697 Å^3^ (CID_9874191); 910,706 Å^3^ (CID_387447), 910,685 Å^3^ (CID_3062316) and 910,704 Å^3^ (control), respectively. Na+ and Cl− ions were used to neutralize the system to reach a 0.15 M molar salt concentration. The CID_9874191, CID_387447, CID_3062316, and control ligands was neutralizing the 77, 9, 78, and 78 Na+ and 69, 7, 69, and 69 Cl− ions, respectively. After constructing the solvated system containing protein in complex with the ligand, the system has been minimized and relaxed using the default protocol introduced within the Desmond module with OPLS_2005 force field parameters^60^. The Nose–Hoover temperature coupling and isotropic scaling method were used to keep NPT ensembles at 310 K and one atmospheric (1.01325 bar) pressure, followed by 100 PS recording intervals with an energy of 1.2. The highest temperature of a Newtonian blood fluid flow has been recorded to be 310.0045 K, while the maximum temperature of a non-Newtonian blood fluid flow has been reported to be 310.007 K^69^. As a result, the 310 k temperature has the strongest impact on system^70^. Total number of atoms was calculated as 84,214 (CID_9874191), 86,782 (CID_387447), 84,178 (CID_3062316), and 84,187 (control).

Maestro v-12.5 was used to make all snapshots of MD simulation. The root-mean-square deviation (RMSD), root-mean-square fluctuation (RMSF), radius of gyration (Rg), solvent-accessible surface area (SASA), protein–ligand contacts (P-L contact), ligand-protein contacts (L-P contact), and hydrogen bond interaction were used to evaluate the stability of the complex structure based on the 500 ns trajectory performance using the simulation interaction diagram (SID) of Desmond module v6.3.

### MM-GBSA analysis from post molecular dynamic simulation trajectory

For calculating the binding free energy of ligands to the macromolecules “molecular mechanics generalized Born surface area” (MM-GBSA) methods have become popular methods^71^. The MM-GBSA has been used to estimate the binding free energy of the compounds by using the Maestro package that incorporated in Schrödinger (Release 2020-3) by Using default parameters.

## Supplementary Information

Supplementary Information.

## Abbreviations

AA: Amino acid
CAAD: Computer-aided drug design
DFT: Density Functional Theory
GLOBOCAN: Global Cancer Observatory
η: Hardness
LYP: Lee, Yang, and Parrs
RO5: Lipinski rule of five
MCM7: Minichromosome Maintenance Complex Component 7
MDS: Molecular dynamic simulation
MM-GBSA: Molecular Mechanics-Generalized Born Surface Area
PDB: Protein Data Bank
P-L contact: Protein–Ligand contacts
QM: Quantum mechanical
Rg: Radius of gyration
RMSD: Root-mean-square deviation
RMSF: Root-mean-square fluctuation
SID: Simulation Interaction Diagram
S: Softness
SASA: Solvent-accessible surface area
TSA: Trichostatin A
