# Supplementary material for: Bioinformatics approaches identified dasatinib and bortezomib inhibit the activity of MCM7 protein as a potential treatment against human cancer
Source: Sci Rep. 2022 Jan 27;12:1539. doi: 10.1038/s41598-022-05621-0 (PMC8795118; doi:10.1038/s41598-022-05621-0)

**Supplementary Materials (SM)**

**Bioinformatics Approaches Identified Dasatinib and Bortezomib Inhibit the Activity of MCM7 Protein as a Potential Treatment Against Human Cancer**

Abdus Samad ^1, 2^, Md. Amdadul Huq ^3,*^, Md. Shahedur Rahman^1, 2, *^

^1^ Department of Genetic Engineering and Biotechnology, Faculty of Biological Science and Technology, Jashore University of Science and Technology, Jashore 7408, Bangladesh

^2^ Bioinformatics and Microbial Biotechnology Laboratory, Department of Genetic Engineering and Biotechnology, Jashore University of Science and Technology, Jashore 7408, Bangladesh

^3^ Department of Food and Nutrition, College of Biotechnology and Natural Resource, Chung-Ang University, Anseong, Gyeonggi-do, 17546, Republic of Korea

*** Corresponding Author**

1. Md. Shahedur Rahman

Email: [ms.rahman@just.edu.bd](mailto:ms.rahman@just.edu.bd)

Department of Genetic Engineering and Biotechnology, Faculty of Biological Science and Technology, Jashore University of Science and Technology, Jashore-7408, Bangladesh

Bioinformatics and Microbial Biotechnology Laboratory, Department of Genetic Engineering and Biotechnology, Jashore University of Science and Technology, Jashore 7408, Bangladesh

2. Md. Amdadul Huq

Email: [amdadbge@gmail.com](mailto:amdadbge@gmail.com)

Department of Food and Nutrition, College of Biotechnology and Natural Resource, Chung-Ang University, Anseong, Gyeonggi-do, 17546, Republic of Korea

**SM 1.** A list of drugs and their Pubchem ID.

| Compounds Name | Pubchem CID |
| --- | --- |
| 2-(2-amino-3-methoxyphenyl)-4H-1-benzopyran-4-one | CID_4713 |
| 2,3-bis(3'-hydroxybenzyl)butyrolactone | CID_123917 |
| 2-amino-1-methyl-6-phenylimidazo(4,5-b)pyridine | CID_1530 |
| 4-(5-benzo(1,3)dioxol-5-yl-4-pyridin-2-yl-1H-imidazol-2-yl)benzamide | CID_4521392 |
| (6-(4-(2-piperidin-1-ylethoxy)phenyl))-3-pyridin-4-ylpyrazolo(1,5-a)pyrimidine | CID_11524144 |
| 7,8-Dihydro-7,8-dihydroxybenzo(a)pyrene 9,10-oxide | CID_41322 |
| Acetaminophen | CID_1983 |
| Aflatoxin B1 | CID_186907 |
| belinostat | CID_6918638 |
| Benzo(a)pyrene | CID_2336 |
| beta-methylcholine | CID_16942 |
| bisphenol A | CID_6623 |
| Bortezomib | CID_387447 |
| Calcitriol | CID_5280453 |
| Carbamazepine | CID_2554 |
| Coumestrol | CID_5281707 |
| cryptolepine | CID_82143 |
| Curcumin | CID_969516 |
| Dasatinib | CID_3062316 |
| decitabine | CID_451668 |
| Diclofenac | CID_3033 |
| Estradiol | CID_5757 |
| Etoposide | CID_36462 |
| Fluorouracil | CID_3385 |
| Genistein | CID_5280961 |
| (+)-JQ1 compound | CID_46907787 |
| K 7174 | CID_9874191 |
| lapatinib | CID_208908 |
| palbociclib | CID_5330286 |
| p-Chloromercuribenzoic Acid | CID_1730 |
| phenethyl isothiocyanate | CID_16741 |
| Phenobarbital | CID_4763 |
| Polychlorinated Biphenyls | CID_6636 |
| resveratrol | CID_445154 |
| Tamoxifen | CID_2733526 |
| Tetrachlorodibenzodioxin | CID_15625 |
| Thapsigargin | CID_446378 |
| trans-10,cis-12-conjugated linoleic acid | CID_5282800 |
| Tretinoin | CID_444795 |
| trichostatin A (Control) | CID_444732 |
| troglitazone | CID_5591 |

**SM 2.** Glide score and binding free energy score of all compounds.

| Entry Name | Docking Score (kcal/mol) | MMGBSA dG Bind(NS) |
| --- | --- | --- |
| CID_123917 | -6.265 | -45.81 |
| CID_5281707 | -6.232 | -41.69 |
| CID_387447 | -6.137 | -56.97 |
| CID_451668 | -5.92 | -46.33 |
| CID_445154 | -5.427 | -35.42 |
| CID_41322 | -5.347 | -31.28 |
| CID_186907 | -5.063 | -33.14 |
| CID_5280961 | -5.057 | -38.86 |
| CID_4713 | -5.013 | -24.69 |
| CID_5330286 | -5.013 | -54.41 |
| CID_82143 | -4.998 | -34.77 |
| CID_208908 | -4.99 | -60.32 |
| CID_1983 | -4.931 | -25.96 |
| CID_3062316 | -4.905 | -50.65 |
| CID_6623 | -4.83 | -27.76 |
| CID_16942 | -4.717 | -25.28 |
| CID_9874191 | -4.578 | -71.85 |
| CID_5757 | -4.536 | -21.25 |
| CID_444732 (control) | -4.529 | -50.11 |
| CID_2554 | -4.47 | -20.38 |
| CID_3385 | -4.452 | -6.04 |
| CID_5280453 | -4.404 | -34.83 |
| CID_969516 | -4.287 | -43.49 |
| CID_4521392 | -4.252 | -53.28 |
| CID_36462 | -4.208 | -49.34 |
| CID_3033 | -4.108 | -27.52 |
| CID_4763 | -4.072 | -34.45 |
| CID_1530 | -4.061 | -32.23 |
| CID_11524144 | -4.015 | -52.01 |
| CID_2336 | -3.962 | -30.67 |
| CID_6636 | -3.893 | -35.06 |
| CID_15625 | -3.688 | -29.1 |
| CID_2733526 | -3.615 | -33.31 |
| CID_5591 | -3.549 | -40.8 |
| CID_444795 | -3.474 | -25.17 |
| CID_446378 | -3.153 | -45.29 |
| CID_46907787 | -2.702 | -29.7 |
| CID_16741 | -2.691 | -22.1 |
| CID_6918638 | -2.656 | -35.5 |
| CID_5282800 | 0.782 | -34.82 |

**SM3.** 2D interaction of MCM7-Drugs. In 3D interaction map, surface is generated based on the type of residue, and in 2D maps, hydrogen bond formation (pink arrows), hydrophobic (green), polar (blue), red (negative), violet (positive), glycine (grey) and π–π stacking (green line) interaction are logged for respective docked complexes.


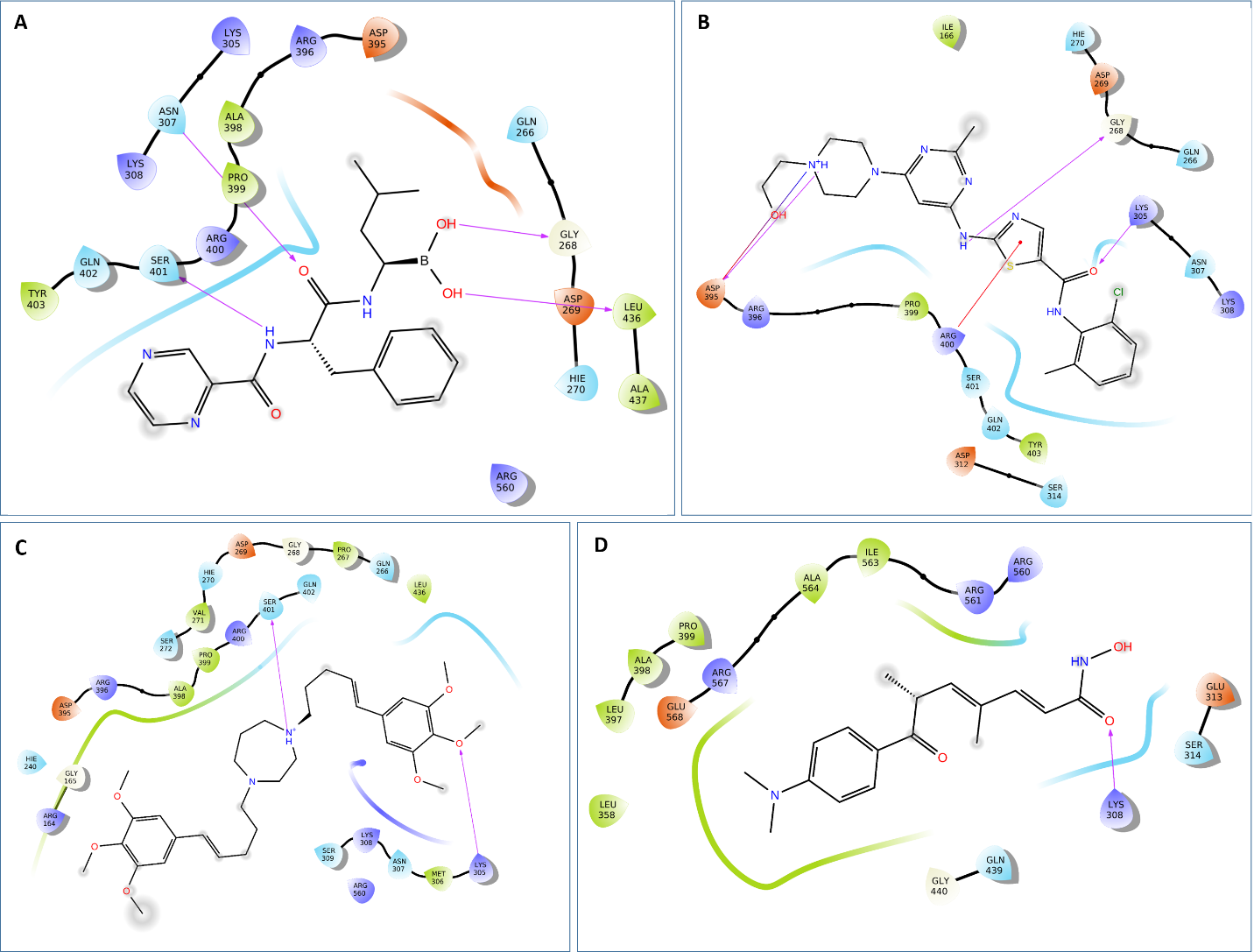

Supplement: Supplementary file 1 — Supplementary Information. [file 41598_2022_5621_MOESM1_ESM.docx]
